# An Insight into Antihyperlipidemic Effects of Polysaccharides from Natural Resources

**DOI:** 10.3390/molecules27061903

**Published:** 2022-03-15

**Authors:** Yong-Shuai Jing, Yun-Feng Ma, Fei-Bing Pan, Ming-Song Li, Yu-Guang Zheng, Lan-Fang Wu, Dan-Shen Zhang

**Affiliations:** 1 College of Chemistry and Pharmaceutical Engineering, Hebei University of Science and Technology, 26 Yuxiang Street, Shijiazhuang 050018, China; cjys1985@126.com (Y.-S.J.); mayunfeng@hebust.edu.cn (Y.-F.M.); limingsong@hebust.edu.cn (M.-S.L.); 2 Huachuang Institute of Areca Research-Hainan, Haikou 570125, China; panfeibing@126.com; 3 College of Pharmacy, Hebei University of Chinese Medicine, 3 Xingyuan Road, Shijiazhuang 050200, China; zhengyg314@126.com

**Keywords:** hyperlipidemia, polysaccharides, structure–activity relationship, mechanisms

## Abstract

Hyperlipidemia is a chronic metabolic disease caused by the abnormal metabolism of lipoproteins in the human body. Its main hazard is to accelerate systemic atherosclerosis, which causes cerebrovascular diseases such as coronary heart disease and thrombosis. At the same time, although the current hypolipidemic drugs have a certain therapeutic effect, they have side effects such as liver damage and digestive tract discomfort. Many kinds of polysaccharides from natural resources possess therapeutic effects on hyperlipidemia but still lack a comprehensive understanding. In this paper, the research progress of natural polysaccharides on reducing blood lipids in recent years is reviewed. The pharmacological mechanisms and targets of natural polysaccharides are mainly introduced. The relationship between structure and hypolipidemic activity is also discussed in detail. This review will help to understand the value of polysaccharides in lowering blood lipids and provide guidance for the development and clinical application of new hypolipidemic drugs.

## 1. Introduction

Diets high in fats and carbohydrates can lead to obesity and hyperlipidemia. Hyperlipidemia is featured by the increase of serum total cholesterol (TC), low-density lipoprotein (LDL), very low-density lipoprotein (VLDL) and the decrease in high-density lipoprotein (HDL) [1]. Hyperlipidemia is regarded as a traditional cardiac risk factor and has been associated with an increased danger of cardiovascular disease among these patients [2]. Therefore, ameliorating hyperlipidemia is significant for preventing and treating cardiovascular and cerebrovascular diseases and reducing social pressure.

Statins are the first-line therapy to reduce LDL levels by inhibiting 3-hydroxy-3-methylglutaryl-coenzymeA (HMG-CoA) reductase. However, statins have side effects, including muscle myopathy and liver dysfunction [3]. At present, other lipid-lowering drugs in clinical practice also include fibrates, niacin, and its derivatives, ezetimibe, etc. Although the lipid-lowering effect is significant, the side effects are also serious, including liver injury, facial flushing, hyperglycemia, high uric acid or gout, and upper gastrointestinal discomfort [4,5,6]. Therefore, finding safer and more effective lipid-lowering drugs is necessary.

Nowadays, an increasing amount of people tend to choose safe and non-toxic naturally derived drugs for disease prevention and medical applications. As a major part of natural resources, polysaccharides have attracted increasing attention [7]. Polysaccharides extracted from natural sources are composed of more than ten monosaccharide molecules connected by different glycosidic bonds, and their molecular structure is complex. In recent years, polysaccharides have been proven to have safe and effective hypolipidemic, antioxidant, liver protection, and immune regulation effects [8]. A great number of studies have reported that polysaccharides have significant curative effects in regulating blood lipids and have broad development prospects. For instance, Rjeibi et al., reported that polysaccharides from *Nitraria retusa* fruits could reduce the hyperlipidemia, hepatotoxicity, cardiovascular, and coronary diseases caused by Triton X-100 [9]. Zhang et al., found that the mechanism of *Pleurotus ostreatus* polysaccharide regulating dyslipidemia is partly related to correcting the abnormal levels of 15 potential biomarkers, such as glycerol phospholipids, aliphatic acids, propylene alcohol lipids, and sphingolipid metabolism [10]. These processes involve different targets and cell signaling pathways and need to be summarized systematically. In addition, it is universally known that the biological activity of polysaccharides is affected by their molecular weight (MW), monosaccharide composition, glycosidic bond type, and sulfate content [11], and their action results are not consistent.

At present, the research summary on the reduction of blood lipids by polysaccharides is not systematic and complete in previous information. Therefore, in this review, we summarize a series of potential pharmacological mechanisms and targets of natural polysaccharides reported in recent years. In addition, the structure–activity relationship is discussed, which has guided the development of new clinical lipid-lowering drugs.

## 2. Lipid Metabolism in the Body

Lipids are one of the most important nutrients needed by the human body. They can provide the energy and essential fatty acids needed by the body and constitute the components of human cells and tissues. Lipids include triglycerides, phospholipids, and sterols. Lipids in the body mainly come from the absorption of exogenous food and endogenous synthesis. The body regulates metabolism through its own mechanism to maintain its dynamic balance.

### 2.1. Digestion and Absorption of Exogenous Lipids

Exogenous lipids are ingested by the body from the diet, including all meat, eggs, animals. The lipids in food cannot be digested in the mouth and stomach of adults. The digestion of lipids is mainly carried out in the small intestine. Firstly, in the upper part of the small intestine, through peristalsis of the small intestine, the bile salts in the bile emulsify the food lipids and render them insoluble. Lipids in water are dispersed into small oil-in-water clusters. At the formed water–oil interface, the enzymes contained in the pancreatic juice secreted into the small intestine begin to digest the lipids in the food. These enzymes include pancreatic lipase, phospholipase, cholesterol esterase, and phospholipase A2. Under the action of enzymes, the micelles are hydrolyzed into glycerol, monoacylglycerol, fatty acid, lysophospholipid, cholesterol, and other small molecules.

The absorption of lipids is mainly in the lower duodenum and cecum. Glycerol and short-chain fatty acids are directly absorbed into the mucosal cells of the small intestine, and then enter the blood through the portal vein. Long-chain fatty acids and other lipid digestion products are absorbed into small intestinal mucosal cells along with micelles. Long-chain fatty acids are catalyzed by acetoacetyl coenzyme A (acyl-CoA) synthase to generate acyl-CoA. Acyl-CoA can esterify monoglycerides, lysophospholipids, and cholesterol to the corresponding triglycerides, phospholipids, and cholesterol esters under the action of transacylase. In the small intestinal mucosal cells, triglycerides, phospholipids, cholesterol esters, and a small amount of cholesterol are produced together with the apolipoprotein synthesized in the cells to form chylomicrons, which eventually enter the blood through the lymph and are used by other cells.

### 2.2. Synthesis, Transport, Absorption, Distribution and Metabolism of Endogenous Lipids

**Figure 1:**
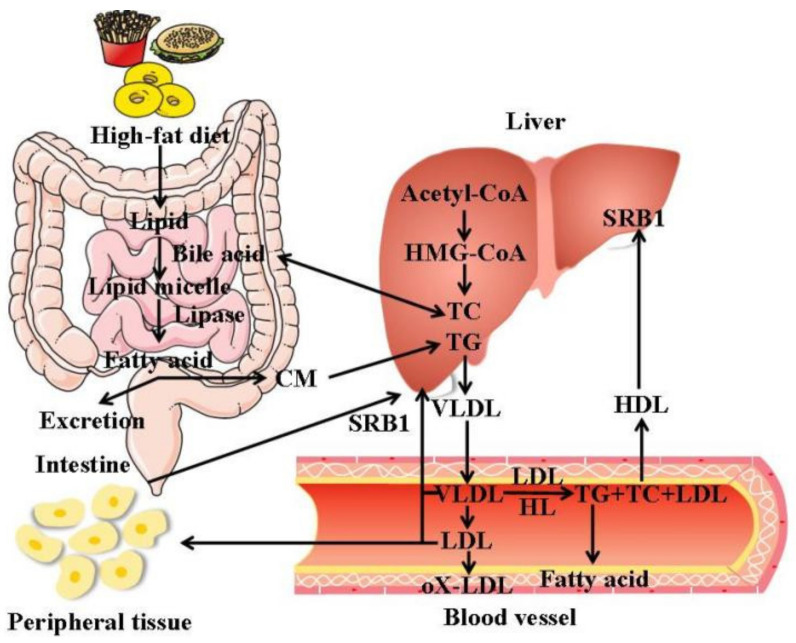
Lipid metabolism in vivo.

The liver, adipose tissue, and the small intestine are important sites for the synthesis of endogenous lipids. The liver has the strongest synthesis ability, but liver cells cannot store fat. After synthesis, it must be combined with apolipoprotein and cholesterol to form very low-density lipoprotein, which is transported into the blood and transported to extrahepatic tissues for storage or use. If the triglycerides synthesized by the liver cannot be transported in time, thefatty liver will be formed. LDL is a kind of lipoprotein particle that carries cholesterol into peripheral tissue cells and can be oxidized to oxidized (OX) LDL. When LDL, especially OX-LDL, is excessive, the cholesterol will accumulate on the arterial wall, which can easily cause arteriosclerosis over time. HDL carries cholesterol in the surrounding tissues, and converts it into bile acids or is directly excreted from the intestines through bile. The lipid metabolism process in vivo is shown in Figure 1.

## 3. Hypolipidemic Effect Mechanisms of Polysaccharides

At present, research evaluating the effect of lowering blood lipids is mainly based on in vitro experiments and animal experiments. In vitro tests include the determination of the lipase inhibition rate and bile salt-binding capacity. In animal experiments, mice were fed a high-fat diet to establish a hyperlipidemia model. After drug treatment, blood lipid-related factors were determined. By consulting the literature, as shown in Table 1, we summarized the source, monosaccharide composition, molecular weight, animal model, and lipid-lowering mechanism of polysaccharides, and described their effects on the absorption, distribution, metabolism, and excretion of TC and triglyceride (TG) in the body. It can be seen from Table 1 that at present, most studies on reducing blood lipids by polysaccharides are carried out at the animal level. At the same time, the means of constructing a hyperlipidemia model is mainly to feed experimental animals with a high-fat diet. In addition, summarizing the lipid-lowering mechanism can provide a theoretical basis and data support for the follow-up study of lipid-lowering polysaccharides.

### 3.1. Inhibit the Absorption of Exogenous Lipids or Promote Cholesterol Excretion

The small intestine can absorb exogenous lipids. After hepatointestinal circulation, most of these lipids will be absorbed and reused by the liver, and a small part will be excreted with feces. Polysaccharides can avoid the hepato-intestinal circulation, reduce reabsorption, promote the excretion of bile acids, and reduce exogenous fat reabsorption. Zhao et al. [12] found that polysaccharides from *Auricularia polytricha* (SPAP) reduced the concentration of blood lipids in serum and brought it close to the normal level. Auricularia polysaccharides can combine with lipid molecules or cholates in the gastrointestinal tract, so as to restrain the absorption of exogenous lipids and boost the metabolism of total cholesterol [13].

### 3.2. Affect Lipid Transport or Distribution

Lipids are insoluble in water and must be combined with lipoproteins to form a water-soluble lipoprotein complex to be transported, mainly with the help of low-density lipoprotein cholesterol (LDL-C) and high-density lipoprotein cholesterol (HDL-C). HDL could transport cholesterol from the peripheral tissues to the liver by the “reverse cholesterol transport” pathway for catabolism, and a high level of HDL had a protective effect [46]. Apolipoprotein (Apo) A is the major apolipoprotein of HDL and an important activator of lecithin cholesterol acyltransferase. It can promote the clearance of free TC in aortic cells and fibroblasts, as well as esterize the free TC in the surrounding tissues and transport it to the liver for metabolism. Therefore, the reverse TC transformation of HDL is achieved by apo A [14]. As the carrier of TC, LDL-C can easily lead to atherosclerotic plaque lesionsif it accumulates too much on the vascular blood wall [16]. ApoB as an LDL receptor is a recognized marker of the LDL receptor. It can transport the LDL into the cell through the LDL receptor on the cell membrane. ApoB is captured by arterial mural cells to initiate and maintain atherogenesis. Wang et al. [47] established an atherosclerotic rat model by feeding a high-fat and high-calcium diet for 30 days. After that, rats were treated with different doses of *Opuntia dillenii* Haw polysaccharides (OPS) intraperitoneally for 60 days. They found that the level of hepatic apoB in rats in the high-dose OPS group was significantly lower compared with that in the model group. This study suggested that OPS might exert anti-atherosclerotic effects by inhibiting apoB protein expression levels. Researchers also found that polysaccharides in Shangluo *Eucommia folium* (EEPs) can obviously decrease the apo B level in blood serum. At the same time, the apo A level in blood serum also increased significantly. This indicates that the blood lipid level can be ameliorated by feeding EEPs, thus reducing the risk of arteriosclerosis and coronary heart disease [48]. Therefore, it can be seen that polysaccharides can promote apoA expression and inhibit apoB expression, improve blood lipid levels in vivo, and thus achieve an anti-atherosclerotic effect.

### 3.3. Affect the Synthesis and Metabolism of Endogenous Lipids

#### 3.3.1. Inhibit the Synthesis of Endogenous Cholesterol

Endogenous cholesterol is mainly synthesized in the liver. By affecting the activity and content of key enzymes in cholesterol synthesis, it inhibits endogenous cholesterol synthesis and reduces blood lipid production. Polysaccharides can competitively inhibit HMG-CoA reductase and limit the synthesis of endogenous cholesterol [49]. Polysaccharides can also reduce TG synthesis by reducing the expression of fatty acid synthase (FAS) mRNA. Zhao et al. [16] found that *Opuntia dillenii* Haw. polysaccharides (ODP-Ia) significantly inhibited the activity of HMG-CoA reductase in the liver of hyperlipidemia rats, thereby affecting the endogenous biosynthesis of cholesterol to prevent nonalcoholic fatty liver disease.

#### 3.3.2. Promote Cholesterol Metabolism

Cholesterol 7α-hydroxylase (CYP7A1) is the rate-limiting enzyme that catalyzes the breakdown of cholesterol into bile acids in the liver and is regulated by multiple factors to maintain the balance of cholesterol metabolism. The protein that secretes bile acids in hepatocytes is the bile salt export pump (BSEP). Polysaccharides can affect the activity and content of these enzymes and proteins related to fat metabolism to regulate cholesterol metabolism. *Grifola frondosa* polysaccharides (GFP) can significantly increase the mRNA expression of cholesterol CYP7A1 and BSEP in hyperlipidemic mice, and enhance the synthesis and excretion of bile acids (BA) in the liver, thereby preventing hyperglycemia and hyperlipidemia in diabetic mice [17]. Lecithin cholesterol acyltransferase (LCAT) can be synthesized in the liver, which is a key factor in maintaining the surface composition of lipoprotein. The release of LCAT into the circulation can catalyze the transfer of long-chain fatty acyl groups from the second position of lecithin to the 3-β-hydroxy group of cholesterol, forming cholesterol ester and lysolecithin [18]. ODP-Ia can significantly increase the serum LCAT activity in hyperlipidemic rats, facilitate normal HDL-C metabolism, and may produce a marked effect in reversing cholesterol transport [16]. LPL can catalyze TG decomposition into fatty acids and monoglycerides and participate in the conversion of apolipoproteins and phospholipids between VLDL and HDL. Oral administration of red ginseng acidic polysaccharide (RGAP) dose-dependently upregulates the LPL activity of hyperlipidemia rats and reduces the level of TG to regulate the hyperlipidemia state of rats [19].

#### 3.3.3. Transcription Factors and Adipokines That Regulate Lipid Metabolism

Polysaccharides can significantly upregulate peroxisome proliferator-activated receptor (PPAR)-α, downregulate PPAR-γ and CCAAT/enhancer-binding protein (C/EBP)-α expression, reduce the level of interleukin (IL)-6, leptin, resistin, and interferon-α (TNF-α), and increase adiponectin levels. Polysaccharides inhibit the differentiation of 3T3-L1 preadipocytes into adipocytes, reduce the expression of PPART and C/EBPa in 3T3-L1 preadipocytes, and inhibit the expression of 3T3 adipocytes involved in lipid synthesis genes and the formation of intracellular lipid droplets. Liu et al. [20] found that the administration of whole *Liriope spicata* var. *prolifera* polysaccharides (TLSP) could significantly reduce the levels of serum TC, TG, and LDL-C in C57BL/6J mice with hyperlipidemia and downregulate PPAR-γ and FAS expression in mouse adipose and liver tissue. TLSP activates lipid/bile acid metabolism through FXH-SHP/CYP7A1 and SEBP-1c/FAC/ACC signaling pathways and plays a role in reducing blood lipid and liver protection. Yu et al. [21] isolated and purified the RLP-1 component from crude polysaccharide of *Rosae Laevigatae Fructus*. The hyperlipidemia rat model was established by a high-fat diet and then treated with RLP-1 for four weeks. The results showed that the levels of TC, TG, and LDL-C in serum of RLP-1-treated rats decreased significantly, but PPAR-γ and LPL were upregulated. Therefore, RLP-1 may play a role in reducing blood lipids by regulating PPAR-mediated lipid metabolism. Hu et al. [22] studied the lipid-lowering effect of *Cyclocarya paliurus* polysaccharides (CPP) on hyperlipidemia rats induced by a high-fat diet (HFD). They found that CPP regulates C/EBP-α, peroxisome proliferator-activated receptor PPAR-γ, PPAR-δ, and c-Cbl-associated protein (CAP) expression, improves glucose tolerance and insulin resistance.

#### 3.3.4. Increase the Expression of LDL Receptor (LDLR) and Accelerate the Decomposition of LDL-C

The liver receptor of low-density lipoprotein (LDL-R) is the main way to mediate LDL clearance. LDL-R functionally defective LDL-R will reducewill reduce the clearance of plasma LDL-C. Purified *Auricularia Auricular* polysaccharide (AAP-I) can stabilize the contents of serum TC, TG, and LDL-C in hyperlipidemia mice at a low level, upregulate LDL-R or gene transcription, and promote the clearance of cholesterol from the circulation [23]. In the HFD with 1% or 5% *Pleurotus eryngii* polysaccharide fraction group, the gene expression of SREBP2 and the mRNA expression of its target gene LDL-R in the liver increased significantly, which increased the uptake of LDL-C in blood and finally caused the decrease in blood lipids [24].

### 3.4. Lower Blood Lipids through Anti-Oxidation

Oxidative stress is a state of imbalance between oxidation and antioxidation in vivo, and it is also one of the important causes of hyperlipidemia and related diseases [50]. Excessive active oxygen free radicals in the body can damage tissues and cells, induce lipid peroxidation, and disrupt lipid metabolism. Polysaccharides can increase the antioxidant capacity of the body and reduce the production of free radicals by increasing the activity of antioxidant enzymes such as glutathione peroxidase (GSH-Px), superoxide dismutase (SOD), and catalase (CAT), and reduce the formation of malondialdehyde (MDA). Polysaccharides can also reduce lipid peroxidation of the cell membrane by removing excess free radicals in the body to reduce blood lipids. Yang et al. [25] found that the treatment of the *Cyclocarya paliurus* polysaccharide fraction (CPP-2) can significantly improve the activities of SOD, total antioxidant capability (T-AOC), and GSH-Px in hyperlipidemia mice, significantly reduce the contents of MDA and lipid peroxide (LPO) and exert anti-hyperlipidemia activity. After the purification of *Enteromorpha prolifera* polysaccharide (EPF2), the activities of endogenous antioxidant enzymes such as SOD, GSH-Px, and CAT in the serum of hyperlipidemic mice increased significantly, and the content of serum MDA decreased significantly, so as to reduce the lipid peroxidation in the serum and achieve the effect of reducing blood lipids [26]. Zheng et al. [27] reported that mycelia zinc polysaccharide from *Pholiota nameko* SW-02 can significantly offset the increased oxidative stress and prevent the occurrence of hyperlipidemia by promoting SOD and T-AOC activities and reducing LPO and MDA levels. This result is consistent with the hypolipidemic effect of the residue polysaccharide of *Cordyceps militaris* SU-12 reported by Wang et al. [28]. Zhang et al. [29] found that *Rosa Laevigata* fruits polysaccharide (RLP) can reduce blood lipid levels such as TC, TG, and LDL, increase serum HDL-C level, increase antioxidant enzyme levels such as SOD, GSH-Px, and CAT, and upregulate fatty acid desaturase 2 (FADS2), acyl-coenzyme A oxidase 3 (ACOX3), and stearyl coenzyme A dehydrogenase-1 (SCD-1) of lipid metabolism and oxidative stress in hyperlipidemic rats. These data indicate that the blood lipid-lowering effect of polysaccharides is closely related to its antioxidant capacity.

### 3.5. Lower Blood Lipids by Regulating Intestinal Microbes

Dietary polysaccharide is transformed into short-chain fatty acids (SCFAs) by gut microbiota in the large intestine [30]. SCFAs could activate the SCFAs receptor GPR43, facilitating leptin secretion and lipolysis, and inhibit adipogenesis in adipose tissue, thereby regulating the fat metabolism [51]. Zhang et al. [31] found that the intervention of *Auricularia auricula* polysaccharide can better enrich several low abundance SCFA and produce bacteria such as Flavonifractor and Clostridium IV to treat hyperlipidemia. Tong et al. [32] found that chitosan (PC) increased the relative abundance of beneficial bacteria such as Prevotella, Oscillibacter, Alloprevotella, Bifidobacterium, and Alistipes in the gastrointestinal tract, and the abundance of these bacteria was negatively correlated with the serum lipid mass spectrum. It can be seen that PC can improve the disorder of lipid metabolism by regulating the microbiota of the gastrointestinal tract. Li et al. [33] reported that the *Grifola frondosa* polysaccharide (GFP) significantly increased the proportion of Helicobacter, Intestinimonas, Barnesiella, Defluviitalea, Ruminococcus, Flavonifractor, and Paraprevotella in the intestinal flora, but decreased the relative abundance of Clostridium-XVIII, Butyricicoccus, and Turicibacter, thereby inhibiting hypercholesterolemia induced by a high-fat diet. Other studies have reported that soy hull polysaccharide (SHP) restored blood lipid levels in rats fed a high-fat-high sucrose diet by increasing the abundance of Bacteroidetes and decreasing the abundance of Firmicutes and Firmicutes [34]. It can be seen that polysaccharides play a role in reducing blood lipids by upregulating the abundance of beneficial bacteria and downregulating the abundance of harmful bacteria in the gastrointestinal tract.

### 3.6. Inflammation Pathways

Fenofibrate and simvastatin can significantly reduce the levels of serum TNF-α, IL-6, IL-8, and other pro-inflammatory factors [52]. Some studies have also shown that polysaccharides have certain anti-inflammatory effects [53]. Therefore, preventing inflammation by inhibiting the activation of pro-inflammatory cytokines may be an effective method for treating hyperlipidemia. Hoang et al. [35] found that the sulfated polysaccharide of *Monostroma nitidum* plays a lipid-lowering role by reducing the mRNA expression of iNOS, TNF-α, IL-6, and IL-8 in palmitate-treated apolipoprotein HepG2 cells in a dose-dependent manner.

### 3.7. AMPK Signal Pathway

Phosphorylated AMPK can inhibit the biosynthesis of glucose, cholesterol, and triglycerides in the liver and promote fatty acid oxidation. There are two important downstream pathways of AMPK related to hyperlipidemia. One is AMPK-HMGCR, in which HMG-CoA could regulate cholesterol biosynthesis through the phosphorylation of AMPK and inhibition of the reductase HMGCR, which was the rate-limiting enzyme. The other is AMPK-ACC-CPT1, in which the phosphorylated AMPK could prevent lipid synthesis and favor fatty acid import into the mitochondria for oxidation [54]. Wu et al. [36] reported that purified polysaccharides from *Cichorium intybus* L. roots may induce fatty acid oxidation and transport by enhancing AMPK activation (FAS↓, adipose triglyceride lipase (ATGL)↑, carnitine palmitoyltransferase-1 (CPT1)↑, acetyl-CoA carboxylase (ACC)↑, and SCD1↓) and reduce lipid biosynthesis to improve fatty liver. Mohammad Raish [55] evaluated the lipid-lowering effect and molecular mechanism of oat β-glutanose (OβG) by using the hyperlipidemia mouse model induced by an HFD and the lipid accumulation model of HepG2 cells induced by oleic acid. The results in vivo and in vitro showed OβG might exert its hypolipidemic activity by inhibiting adipogenesis by activating the AMPK signaling pathway.

### 3.8. Polysaccharide Improves Iinsulin Rresistance

**Figure 2:**
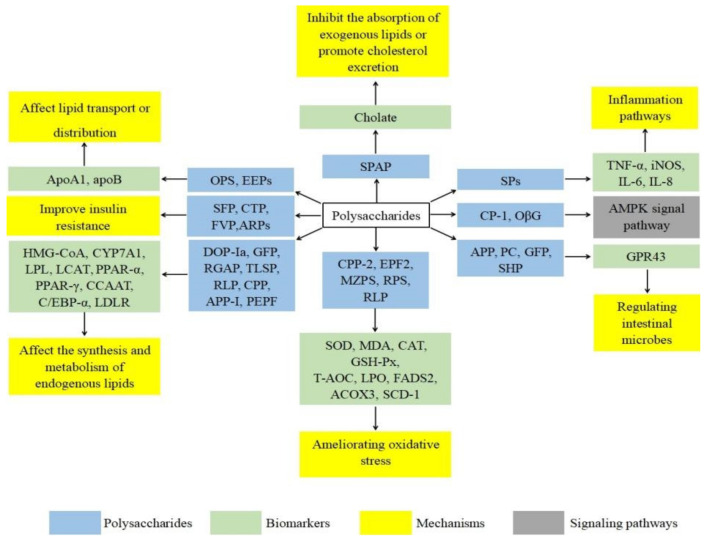
Polysaccharides and its various mechanisms for lowering blood lipids.

Type II diabetes is a complex hyperglycemia metabolic syndrome that mainly reduces insufficient insulin secretion and insulin resistance [56]. The liver is regarded as a vital organ for metabolic balance, as it is responsible for glucose and lipid metabolism [57]. Sustained hyperglycemia, as is well-known, is always accompanied by theaccumulation of TC, TG, and LDL-C as well as a decrease in HDL-C, leading to the occurrence of hyperlipidemia [58]. Therefore, the effective prevention and treatment of hyperglycemia can reduce the occurrence of hyperlipidemia diseases. Although hyperglycemia and hyperlipidemia are inseparable, the mechanism of lipid metabolism disorders in diabetes remains unclear and requires close study. Jia et al. [37] induced hyperglycemia and hyperlipidemia in SD rats by an HFD and injection of streptozotocin solution. Sargassum polysaccharides can promote the transport of TC and TG from plasma to the liver by reducing the level of serum LDL-C and significantly improving hyperglycemia, hyperlipidemia, liver, and kidney function of diseased rats. In another study, Liu et al. [38] reported that *Cordyceps taii* polysaccharide can upregulate insulin secretion, increase glucose uptake, inhibit hormone-sensitive lipase, reduce free fatty acids (FFA), and reduce blood lipid levels in hyperglycemic mice. Song et al. [39] also found that probiotic-fermented milk containing *Flammulina velutipes* polysaccharide (FVP) could reduce the levels of TC, TG, LDL-C, and FFA and increase the HDL-C levelin diabetic mice. Meanwhile, it was found that probiotic-fermented milk containing FVP could regulate dyslipidemia and glycometabolism disorder through the PI3K/Akt signal pathway. A study confirmed that *Anoectochilus roxburghii* (Wall.) Lindl. polysaccharides (ARPs) promote fat thermogenesis in part through the AMPK/SIRT1/PGC-1α signaling pathway, thereby promoting energy metabolism and ameliorating the role of glucose and lipid metabolism disturbances in diet-induced obesity [40]. Figure 2 discusses 25 polysaccharides derived from natural sources in recent years and their various mechanisms for lowering blood lipids.

## 4. Structure–Antihyperlipidemic Activity Relationship

**Figure 3:**
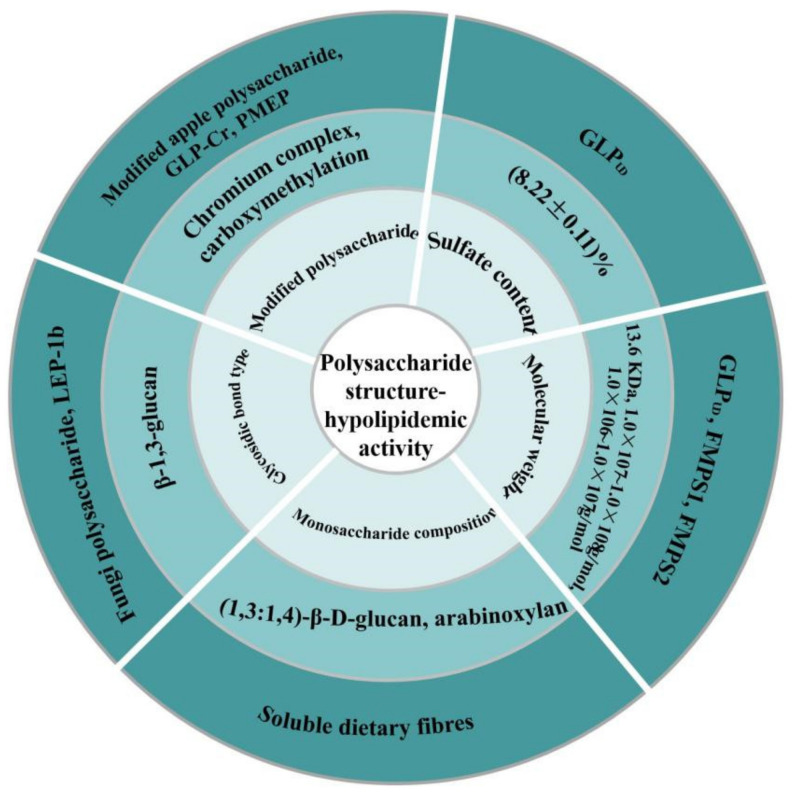
Polysaccharide structure–antihyperlipidemic activity relationship.

The lipid-lowering properties of polysaccharides are closely related to their monosaccharide composition, molecular weight, chain conformation, type and position of glycosidic bond, sulfate content, and other chemical structures. Figure 3 is a summary of the influencing factors of polysaccharide structure and hypolipidemic activity, and examples of related polysaccharides are demonstrated.

Many reports have confirmed that the sulfate content and molecular weight of polysaccharides surprisingly affect their lipid-lowering activity. In the study of hypolipidemic activity of natural polysaccharides and degraded polysaccharides from *Ganoderma lucidum*, researchers found that GLP_UD_ with low molecular weight and an increased number of sulfate groups had higher hypolipidemic activities [41]. Four polysaccharide components from *Fortunella margarita* (Lour.) Swingle polysaccharides (FMPs) were isolated and their binding ability to bile acids was discussed. It was found that FMPS1 and FMPS2 with higher molecular weights bound more bile acids than FMPS3 or FMPS4 [42]. The higher the molecular weight of the polysaccharide, the higher the viscosity. High molecular weight pectin will form a composite gel, increasing its viscosity in the gastrointestinal tract, thereby limiting the diffusion of lipids and lipases, while reducing the overall lipolysis reaction. Finally, it can reduce blood lipids [59,60].

The composition of monosaccharides is closely related to the hypolipidemic activity of polysaccharides. According to reports, (1,3:1,4)-β-D-glucan and arabinoxylan can limit the reabsorption of bile from the ileum into enterohepatic circulation, bile salts enter the colon and are then excreted, supplementing plasma cholesterol to further synthesize vile saltsin order to reduce circulating levels of TC and LDL-C [61].

The anti-hyperlipidemic activity of polysaccharides is also correlated with the type and position of glycosidic linkages. The main chain of lipid-lowering polysaccharides derived from edible fungi (*Pholiota nameko*, *Pleurotus ostreatus*) and non-edible fungi (*Botryosphaeria rhodiua* MAMB-05) are generally β-1,3-glucan and contain a certain number of side chains linked by β-1,3-glucan [62,63]. Qiu et al. [64] isolated a glucan LEP-1b with significant blood lipid-lowering activity from the extracellular polysaccharide of *Lachnum* YM281. Its main chain was composed of β-(1,3)-D-glucan and the LEP-1b had a triple helix conformation.

Some natural polysaccharides have no biological activity or very low activity and low solubility, affecting their biological activity. Therefore, researchers use chemical modification, biological modification, physical modification, and other methods to modify the structure of polysaccharides to change their biological activity and reduce toxicity [43]. Researchers found that modified apple polysaccharidescan reduce HFD-induced obesity in mice, reduce lipid accumulation in adipose tissue and liver, and ameliorate the blood lipid level [65]. Studies have shown that the *Ganoderma lucidum* polysaccharide chromium (GLP-Cr) (III) complex can significantly reduce the levels of serum TC, TG, and LDL-C in prediabetic mice induced by high fructose and a high-fat diet. Further studies found that the mechanism of the GLP-Cr (III) complex regulating blood lipids may be related to the regulation of intestinal microbiota, glucose, and lipid metabolism-related genes [44]. Li et al. [45] compared the serum cholesterol activity, bile acid expression, and interaction of protein expression on cholesterol metabolism between purified *Morchella angusticepes* Peck polysaccharide (PMEP) and chemically carboxymethylated PMEP (CPMEP). The results showed that CPMEP had relatively strong cholesterol-lowering activity. COMEP has the trend of reducing total cholesterol in liver and increasing total bile acid excretion in feces and intestine, which may be mediated by downregulating HMG-CoA reductase and upregulating CYP7A1. Therefore, CPMEP can enhance the ability to reduce cholesterol in rats. It can be seen that the structural characteristics of polysaccharides such as monosaccharide composition, molecular weight, and glycosidic bond types are closely related to the blood lipid-lowering effect of polysaccharides.

## 5. Application of Polysaccharides in Lowering Blood Lipids

At present, diet therapy is mainly used for hyperlipidemia. The diet is mainly low-fat and low-sugar foods. When it is invalid, lipid-lowering drugs such as statins, fibrates, niacin and its derivatives, fish oil preparations, and antioxidant preparations can be appropriately added. Since some lipid-lowering drugs can lead to side effects such as muscle myopathy, liver dysfunction, hyperglycemia, high uric acid or gout, and upper gastrointestinal discomfort, natural products play a distinct and important role in the guidance and reference of new drug development. As naturally active ingredients, polysaccharides have been reported successively to lower blood lipids [66]. Health products are generally called dietary supplements. The application of polysaccharides in lowering blood lipids is mainly focused on the development of healthy foods and health products that regulate blood lipids. Currently, there are many kinds of polysaccharide health products and foods with hypolipidemic effects, including Xinlu Brand Ganoderma Solid Fungal Polysaccharide Capsules, Guangxia Brand Lycium Barbarum Polysaccharide Oral Liquid, Panzhibao Capsules, Baoshengtai, Pengyao Yinling Capsules, and Shiitake Mushrooms Polysaccharide drinks, Yiyuan brand propolis Ganoderma lucidum spore soft capsule, Weihong brand glycolipid light oral liquid, Feide brand Haiyuezhi capsules, Qingchunbao brand spirulina capsules, Qinghong brand Qingqing capsules, and Qingzhiyuan R Highland barley ginkgo capsules. These health products and foods can not only regulate blood lipids, avoid the adverse reactions of blood lipid-lowering drugs, but also improve immunity. The added excipients are safe and non-toxic, and there is no dosage requirement. Therefore, they can be used as a dietary supplement to lower blood lipids but cannot completely replace blood lipid-lowering drugs.

## 6. Conclusions and Further Perspective

At present, there are many side effects from anti-hyperlipidemic drugs on the market, and natural polysaccharides have attracted worldwide attention because of their safety and effective lipid-lowering activity. Therefore, this paper reviews the process of lipid metabolism, the mechanism and structure–activity relationship of natural polysaccharides with lipid-lowering effects in recent years, and polysaccharides with lipid-lowering effects of finished drugs, in order to provide a theoretical basis for the development and application of these natural products in both the functional food and medical industry.

However, most of the reported studies on the lipid-lowering effect of polysaccharides are carried out in in vitro tests or HFD-induced animal models, which cannot fully represent their actual effect on the human body. Therefore, more in-depth clinical research is needed to study the practical application of natural polysaccharides in the human body. In addition, at present, studies have been reported on the primary structure of monosaccharide composition, relative molecular weight, glycoside bond link, but the study of advanced structures, such as the spatial structure, is still very rare, let alone the relationship between structure and function. Therefore, it is necessary to study the molecular structure, active group, physicochemical properties, and structure–activity relationship of polysaccharides. Besides, the mechanism of polysaccharide lowering blood lipidis not clear and is at the speculative stage; therefore, it is necessary to deepen the research, based on existing research, at the biochemical, cell, and gene level to clarify the relevant pathways and mechanisms. Finally, most of the studies on the hypolipidemic activity of polysaccharides are at the laboratory research and development stage, and there are not many related products on the market. It is not yet possible to transform the research results into healthy foods and even drugs that can be produced and sold. Therefore, it is necessary to strengthen the research on their development, utilization, and production technology.

In short, natural sources of polysaccharides can provide good raw materials for the development of blood lipid-lowering health foods and medicines due to their blood lipid-lowering activity. It is necessary to further study them in order to uncover the important value of polysaccharides in the health food industry and in biomedicine.

## Figures and Tables

**Table 1:** Summary of the hypolipidemic effects of natural polysaccharides.

No.	Compound Name	Polysaccharides Source	Molecular Weight (kDa)	Monosaccharide Composition	Experimental Model	Involved Mechanism	Reference
1	NRFP	*Nitraria retusa* fruits	66.5	Ara, Gal, GalUA, Glu, Rha	Swiss albino male mice was induced by intraperitoneal injection of Triton X-100	TC ↓, TG ↓, LDL ↓, HDL-C ↑ ∙Antioxidant activity	[9]
2	POP	*Pleurotus ostreatus*			Male Wistar rats fed with fat emulsion	Fatty acids induced lipotoxicity ↓ Regulate the dysfunction of prenol lipids metabolism and sphingolipids metabolism	[10]
3	SPAP	*Auricularia polytricha*			High-fat and high-cholesterol diet rat	The absorption of exogenous lipids ↓ ∙Total cholesterol metabolism ↑	[12,13]
4	AJP	*Apostichopus japonicus*	36.2	Fuc, Gal, GalN, GlcN, GlcUA, Glu, Man	Male albino rats of Wistar strain fed with high-fat diets	Transport and excretion of serum lipids ↑ Bile acid sequestrant mechanism Antioxidant activity	[14]
5	SMP	*Shiitake* mushroom			Kunming mice fed by high cholesterol diet	Lipid metabolism ↑ Oxidative damage ↓	[15]
6	ODP-Ia	*Opuntia* *dillenii* Haw.	60	Ara, AraUA, Gal, Glu, Rha	Male Sprague-Dawley rats were fed a high-fat emulsion diet	LCAT ↑, HMG-CoA ↓ Antioxidant activity Lipid accumulation ↓, inflammatory cell infiltration ↓	[16]
7	GFP	*Grifola frondosa*	15,850, 280.7, 18.18	Fuc, Gal, GalUA, GlcUA, Glu, Man, Rha	Kunming mice fed with a high-fat diet	Alter gut microbiota and regulate hepatic glycolipid metabolism related genes	[17]
8		*Sargassum polycystum*			Male Wistar strain albino rats were intoxicated with acetaminophen	LCAT ↑ The intestinal absorption of cholesterol ↓, the cholesterol excretion↑ Maintain calcium homeostasis Severe fat changes ↓, inflammation ↓, the levels of HTGL↓ Antioxidant activity	[18]
9	RGAP	Red ginseng acidic			Induced in the male Sprague-Dawley rats with Triton WR1339 or corn oil	The degradation enzyme activity of lipoprotein↑	[19]
10	TLSP	*Liriope spicata* Var. *Prolifera*			C57BL/6J mice with high fat diet	Inhibite PPAR γ2 and the SREBP-1 pathway The LXR/FXR-SHP/CYP7A1 signaling pathway Bile acid metabolism ↑, cholesterol content ↓ Antioxidant activity	[20]
11	RLP-1	*Rosae Laevigatae Fructus*	21.5	Gal, Man, Xyl	Male SPF Sprague-Dawely rats were fed with high-cholesterol diet	Regulate PPAR-mediated lipid metabolism	[21]
12	CPP	*Cyclocarya paliurus*	190.1, 2.1	Ara, Gal, Glu, Man, Rha, Xyl	Female Sprague-Dawley rats were fed with high-fat diet	∙Regulate the activities of hepatic lipid metabolism-related enzymes Insulin resistance↓	[22]
13	AAP-I	*Auricularia auricular* mycelium			Male Kunming mice were fed cholesterol-enriched diet	LDL ↓ Affect gene transcription The removal of cholesterol from circulation ↑	[23]
14	PEPF	*Pleurotus eryngii*			Male C57BL/6J mice was fed with 36% fat diet	Excretion of bile acids and lipids Alter gut microbiota	[24]
15	CPP-2	*Cyclocarya paliurus*	307 3.7	Gal, Glu, Man Rha	Female ICR mice was perfused high-fat emulsion alternated with distilled water	TC ↓, TG ↓, HDL-C ↑, LDL-C ↓ ∙SOD ↑, T-AOC ↑, GSH-PX ↑, MDA ↓, LPO ↓	[25]
16	EPF2	*Enteromorpha prolifera*	103.51	Gal, Glu, Man, Rha, Xyl	Male kunming mice was fed with high-fat diet	Antioxidant activity	[26]
17	MZPS	*Pholiota nameko* SW-02	36.4	Ara, Gal, Glu, Man	Male kunming mice was perfused high-fat emulsion alternated with distilled water	Antioxidant activity	[27]
18	RPS	*Cordyceps militaris* SU-12	2.86	Ara, Glu, Man	Male kunming mice was perfused high-fat emulsion alternated with distilled water	Oxidative stress ↓	[28]
19	RLP	*Rosa Laevigata* fruits			Eight-week-old male rats were fed with high-fat diet and treated with 5% acacia gum solution	FADS2 ↑, ACOX3 ↑, SCD-1 ↑	[29]
20		*Chenopodium quinoa* Willd.	82.7	Ara, Gal, GalUA, GlcUA, Glu, Man, Rha, Xyl	SPF Sprague-Dawley rats were fed with high-fat diet	Affect the gut microbial composition	[30]
21	AAP	*Auricularia auricula*			Male Sprague-Dawley rats were fed with high-fat diet for 4 weeks	Regulation of the gut microbiota structure	[31]
22	PC	Chitosan			Male Syrian golden hamsters were fed with high-fat diet	Modulate gastrointestinal microbiota	[32]
23	GFP	*Grifola frondosa*			Male 6-week-old Wistar rats were fed with high-fat diet	∙Modulate specific gut microbial phylotypes ∙Regulate hepatic lipid and cholesterol metabolism related genes	[33]
24	SHP	Soy hull			Male SD rats were fed high-fat-high-sucrose diet	The abundance of Bacteroidetes↑, the abundance of Firmicutes and Firmicutes↓	[34]
25	MF	*Monostroma nitidum*			HepG2 cells were cultured for 24 h in DMEM containing 10% FBS, 1% penicillin/streptomycin, and palmitate that was conjugated to 0.16% fatty acid-free BSA	Inflammation pathways	[35]
26	CP-1	*Cichorium intybus* L.	8.5114	Fru, Glu, Sbt, Sor	Five-week-old male Sprague-Dawley rats were fed a high fat diet	Activate AMPK pathway	[36]
27	SFPs	*Sargassum* *fusiforme*	SFP-1: 8.47, 4.33 SFP-2: 84.99, 14.33	∙SFP-1: Gal, GlcUA, Glu, Man ∙SFP-2: Fuc, Gal, GlcUA, Man	Male SD rats were fed with high sugar and fat diets	The serum LDL-C ↓, the transportation of TC and TG from plasma to liver ↑	[37]
28	CTP	*Cordyceps taii*			KM mice were injected with STZ at a dose of 100 mg/kg	Insulin secretion↑, glucose uptake↑, hormone sensitive lipase↓, free fatty acids↓, blood lipid level↓	[38]
29	FVP	*Flammulina velutipes*			Male ICR mice were injected with 100 mg/kg STZ solution once a day, and administrated with high fat and high sugar feed	TC, TG, LDL-Cand FFA↓, HDL-C↑ PI3K/Akt signal pathway	[39]
30	ARPs	*Anoectochilus roxburghii* (Wall.) Lindl.		L-Ara, L-Rha, D-Gal, D-Man, D-Xyl, D-Glu, GalUA, GlcUA, Ribose, Fuc	C57BL/6J male mice were fed a high-fat diet	AMPK/SIRT1/PGC-1α signaling pathway, ameliorating the role of glucose and lipid metabolism disturbances	[40]
31	GLP GLP_UD_	*Ganoderma lucidum*	3.06 × 10^3^ 13.6	Fuc, Gal, GalUA, GlcUA, Glu, Man, Rha, Xyl	Male Kunming mice were fed a high-fat diet for 30 consecutive days	Atherosclerosis index ↓, TC ↓, TG ↓, LDL-C ↓, HDL-C↑ ∙Antioxidant activity	[41]
32	FMPS	*Fortunella margarita* (Lour.) Swingle		∙FMPS1: Gal, GalUA, Glu, Man, Rha ∙FMPS2: Ara, Gal, GalUA, Glu, Man ∙FMPS3: Ara, Gal, GalUA, Man, Rha ∙FMPS4: Gal, GalUA, Man, Rha	In vitro test	The pancreatic lipase activity ↓ Bind bile acid ↑ Antioxidant activity	[42]
33	U/PU	*Ulva pertusa*		U: Glu, GlcUA Rha, Xyl PU: Rha, Xyl	Male Kunming mice were fed a high-fat diet	TC ↓, TG ↓, LDL-C ↓, HDL-C ↑	[43]
34	GLP-Cr(III)	*Ganoderma lucidum*			Male Kunming mice were fed with a high-fructose and high-fat diet	Regulate gut microbiota, and glucose and lipid metabolism related genes	[44]
35	PMEP/CPMEP	*Morchella angusticepes* Peck			Sprague-Dawley rats were fed the high cholesterol diet	CYP7A1 ↑, LDL-R ↑, HMG-CoA ↓	[45]

Note: Ara: arabinose, AraUA: arabinuronic acid, Fru: fructose, Fuc: fucose, Gal: galactose, GalN: galactosamine, GalUA: galacturonic acid, GlcN: glucosamine, GlcUA: glucuronic acid, Glu: glucose, Man: mannose, Rha: rhamnose, Rib: ribose, Sbt: Sorbitol, Sor: sorbin, Xyl: xylose; ↑: upregulate; ↓: downregulate.
